# Ohio State Dental Society

**Published:** 1890-10

**Authors:** 


### Ohio State Dental Society.

From reliable information, the statement is fully warranted that preparation is being made for an interesting meeting, of this, one of the oldest State dental societies in the country.

The programme will soon be issued, and will be a good one. There will be a number of papers on interesting topics by men both inside and outside the State, and these will doubtless draw out much good discussion.

A half a day at least, will be set apart for clinics, demonstrations, and illustrations.

The latter will be demonstrated by the use of the micro-lantern and its accessories; this will be done by some of the best workers in this line in the country.

There will be many new and interesting instruments and appliances presented.

The promise for a good meeting is so encouraging that we feel like urging all to be present, and so far as possible to take part in the proceedings.

